# Screening for lung cancer: A systematic review of overdiagnosis and its implications

**DOI:** 10.1002/1878-0261.70139

**Published:** 2025-11-11

**Authors:** Fiorella Karina Fernández‐Sáenz, Laura de la Torre‐Perez, David R Baldwin, Carlijn van der Aalst, Mangesh Thorat, David Ritchie, Andre L Carvalho, Carolina Espina, Ivan Solà, Carlos Canelo‐Aybar, Moira Magdalena Pissinis, Pablo Alonso‐Coello, Ana Carolina Pereira Nunes Pinto

**Affiliations:** ^1^ Institut de Recerca Sant Pau (IR SANT PAU) Barcelona Spain; ^2^ Centro Cochrane Iberoamericano Barcelona Spain; ^3^ Universidad Peruana Cayetano Heredia Lima Peru; ^4^ Nottingham University Hospitals NHS Trust UK; ^5^ Department of Public Health Erasmus MC, University Medical Center Rotterdam Netherlands; ^6^ Wolfson Institute of Population Health Quenn Mary University of London UK; ^7^ Homerton University Hospital London UK; ^8^ International Agency for Research on Cancer (IARC/WHO) France; ^9^ CIBER de Epidemiología y Salud Pública Instituto de Salud Carlos III Madrid Spain

**Keywords:** low‐dose computed tomography, lung cancer, overdiagnosis

## Abstract

Low‐dose computed tomography (LDCT) screening is increasingly used for early lung cancer detection targeted to high‐risk populations. Quantifying overdiagnosis, its potential harms, and economic consequences is important. We assessed the magnitude, harms, and economic impact of lung cancer overdiagnosis from LDCT screening in high‐risk populations. We synthesized evidence from eight randomized trials involving 84,660 participants. LDCT may increase overdiagnosis compared to no screening (relative risk [RR] 1.05; 222 additional cases per 100 000 people screened; low certainty). Compared to chest x‐ray (CXR), LDCT likely slightly increases overdiagnosis (RR 1.01; 63 additional cases per 100 000 people screened; moderate certainty). The proportion of overdiagnosed cancers is 0.07 (7000 more lung cancers overdiagnosed per 100 000 lung cancers detected; low certainty) when compared to no screening, and 0.01 compared to CXR (1000 more lung cancers overdiagnosed per 100 000 lung cancers detected; moderate certainty). In terms of cost, LDCT resulted in an additional societal burden of €2,026,422.00 per 100 000 individuals screened compared to no screening. The magnitude of overdiagnosis in LDCT screening is likely low compared to CXR.

## Graphical abstract

Low‐dose computed tomography (CT) screening for lung cancer may increase overdiagnosis compared to no screening, though the risk is likely low versus chest X‐ray. Our review of 8 trials (84 660 participants) shows added costs. Further research with strict adherence to modern nodule management strategies may help determine the extent to which overdiagnosis can be reduced while maintaining the benefits of screening.

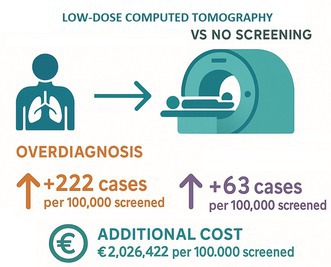

**Abbreviations**
BAC: bronchioloalveolar carcinoma
CENTRAL: cochrane central register of controlled trials
CHEERS: consolidated health economic evaluation reporting standards
CI: confidence interval
CPGs: clinical practice guideline
CT: computed tomography
CXR: chest X‐ray
DANTE: detection and screening of early lung cancer by novel imaging technology trial
DLCST: danish lung cancer screening trial
ECAC5: European Code Against Cancer, 5th edition
EMBASE: excerpta medica database
EQ‐5D VAS: EuroQol 5 dimension visual analogue scale
EU: European Union
FEV1: forced expiratory volume in 1 s
GDP: gross domestic product
GRADE: grading of recommendations, assessment, development and evaluation
HaDEA: health and digital executive agency
HRQoL: health‐related quality of life
IARC: international agency for research on cancer
ICER: incremental cost‐effectiveness ratio
ISPOR: International Society for Pharmacoeconomics and Outcomes Research
JHCS: Johns Hopkins Comparative Study
kV: kilovolts
Kvp: kilovoltage peak
LDCT: low‐dose computed tomography
LLP: liverpool lung project
LSS: lung screening study
LUSI: lung cancer screening intervention trial
mAs: milliamperes
MD: mean difference
MEDLINE: medical literature analysis and retrieval system online
MILC: microsimulation model of the natural history of lung cancer
MILD: multicentric italian lung detection trial
MRI: magnetic resonance imaging
NA: not applicable
NCI: National Cancer Institute
NELSON: Nederlands‐Leuvens Longkanker Screenings Onderzoek trial
NICE: National Institute for Health and Care Excellence
NIESH: National Institute of Environmental Health Sciences
NLST: National Lung Screening Trial
NPC: National Pharmaceutical Council
NR: not reported
OHAT: office of health assessment and translation
PET: positron emission tomography
PRISMA: preferred reporting items for systematic reviews and meta‐analysis
PROSPERO: international prospective register of systematic reviews
RCT: randomized controlled trial
RoB 2: risk of bias tool, version 2
RR: risk ratio
SD: standard deviation
SF‐12: short‐form 12 health survey
SIFs: significant incidental findings
STAI: state–trait anxiety inventory
T2: timepoint 2
UK: UKLS
VAS: visual analogue scale
WHO: World Health Organization

## 1 Introduction

Lung cancer is the leading cause of cancer‐related morbidity and mortality worldwide, with nearly 2.5 million new cases and over 1.8 million deaths annually [1]. Screening with low‐dose computed tomography (LDCT) has been shown to detect lung cancer at earlier, potentially more treatable stages. A systematic review of randomized clinical trials has shown mortality reductions in lung cancer of 14% and all‐cause mortality of 4% [2, 3]. However, early detection does not always translate into improved clinical outcomes, as some detected cancers are indolent and would never have caused symptoms or affected a patient's lifespan. This phenomenon, known as overdiagnosis, is a key concern in screening programs, as it leads to unnecessary treatments, psychological distress, and increased healthcare costs [4].

Overdiagnosis occurs when screening identifies malignancies that would have remained asymptomatic or progressed too slowly to impact a patient's health [4, 5]. This issue is particularly challenging in lung cancer, where LDCT often identifies small lesions (< 1 cm), many of which are benign and a few that are malignant but that may never become clinically relevant [6]. At the same time, lung cancer often progresses relatively rapidly compared to other cancers like colorectal or gastric [7], a duality that demands accurate clinical management that rapidly diagnoses harmful cancer while avoiding overdiagnosis. A sufficiently long post‐screening follow‐up period is crucial to observe a “catch‐up” in diagnoses within the non‐screened population and help determine whether early‐detected cancers in the screened group reflect lead‐time effect or true overdiagnosis [3, 8, 9, 10].

Beyond clinical implications, overdiagnosis has considerable psychological and economic consequences due to unnecessary diagnostic procedures (e.g., follow‐up imaging, biopsies), overtreatment (e.g., surgery, radiation, chemotherapy), and extended patient monitoring [11, 12]. The financial burden of overdiagnosis not only affects individual patients but also places strain on healthcare systems by diverting resources from more urgent needs. In the context of lung cancer, the economic implications of screening have gained traction [12, 13, 14]; however, few have comprehensively evaluated the financial impact attributable to overdiagnosis.

Despite the growing adoption of LDCT screening, high‐quality systematic reviews that simultaneously assess the magnitude of overdiagnosis, its associated harms, and economic impact remain scarce. Addressing this gap is crucial to support informed policy‐making in lung cancer screening programs. This systematic review was undertaken in the context of the update of the European Code Against Cancer, 5th edition (ECAC5) project [15]. It aims to quantify the extent of overdiagnosis from LDCT screening, evaluate the potential clinical harms associated with unnecessary diagnoses and treatments, and estimate the financial burden of overdiagnosis compared to no screening or chest X‐ray (CXR).

## 2 Materials and methods

We performed a systematic review to assess the magnitude and harms of LDCT screening following rapid review guidance developed by the Cochrane Rapid Reviews Methods Group [16] and adhered to the Preferred Reporting Items for Systematic Reviews and Meta‐Analysis (PRISMA) statement [17]. This rapid review was guided by a protocol reviewed by the guideline panel and registered in PROSPERO (CRD42025641923).

To evaluate the economic impact of overdiagnosis, we followed the recommendations of the National Institute for Health and Care Excellence (NICE) for incorporating economic evaluation in clinical practice guidelines (CPGs) handbook, the GRADE working group guidance, and reported this in accordance with the Consolidated Health Economic Evaluation Reporting Standards 2022 [18].

### 2.1 Eligibility criteria and searches

We included randomized controlled trials (RCTs) involving adults at higher risk of lung cancer (e.g., as determined by their history of smoking tobacco and age or by a multivariable risk‐prediction model), using volumetric‐based or diameter‐based screening performed in any periodicity. To assess the economic impact of overdiagnosis, we included any type of study (e.g., piggyback clinical studies, cost‐effectiveness studies) reporting costs associated with overdiagnosis due to LDCT screening, either direct (e.g., resource use or cost of illness studies) or indirect (e.g., burden of disease studies or other study designs quantifying resource use as a secondary objective) in organized population screening programs in European countries. Based on previous modeling studies, we defined overdiagnosis as persisting excess incidence after a follow‐up with no screening for at least 6 years, a duration considered sufficient for a “catch‐up” to occur in the non‐screened group [19, 20]. Following the guidance provided by the Cochrane Rapid Reviews Methods Group [16], we limited the review to articles in peer‐reviewed journals, so we did not consider gray literature or conference abstracts. We excluded studies not published in English.

We performed comprehensive searches on May 15, 2024, in Medical Literature Analysis and Retrieval System Online (MEDLINE), Excerpta Medica Database (Embase), and Cochrane Central Register of Controlled Trials (CENTRAL) databases (see the full search strategy in the Table S1). To identify additional completed or ongoing trials that could be eligible for future inclusion in this review, we also searched the Clinicaltrials.gov platform. Finally, to ensure no further relevant studies were missing, we examined the reference lists of included RCTs.

### 2.2 Data collection, critical appraisal and synthesis of results

Two authors screened search results based on the title and abstract and then on full‐text assessment to identify potentially eligible reports. One reviewer extracted data and assessed the risk of bias of included studies using version 2 of the Cochrane risk‐of‐bias tool for randomized trials (RoB 2) [21] and the ISPOR checklist [22], and a second reviewer cross‐checked the data for accuracy and consistency [16]. Disagreements were solved through consensus.

For all healthcare questions (magnitude, harms, and costs of overdiagnosis), we presented the main results in tabulated summaries. For the results on harms and costs, we calculated their absolute risks associated with overdiagnosis in R (R Core Team 2023) [23].

To estimate the magnitude of overdiagnosis, we pooled results into meta‐analyses to calculate both overdiagnosis from a public health perspective and from a clinical perspective. For the first, we calculated the risk ratio (RR) of lung cancer in the LDCT versus the not screened or other modalities groups. For the overdiagnosis rates under the clinical perspective, we estimated the risk that a screen‐detected lung cancer is overdiagnosed (i.e., what is the likelihood that the lung cancer is overdiagnosed should a person be detected with a cancer in the LDCT screening arm). To do so, we first calculated the diagnosis rate in the screened group and then bootstrapped this to obtain 95% normal‐based confidence intervals, using the Jupyter interface for Python. Where appropriate, we pooled effect sizes (e.g., RR) using a random effects model with the inverse variance method using Revman 5.4.

For overdiagnosis‐related harms, we used the magnitude of overdiagnosis estimate provided by the present review to calculate the absolute risk of harms, based on the incidence of harms in the included trials. To estimate the cost associated with overdiagnosis, we used the magnitude of overdiagnosis estimate provided by our metanalysis, along with the incidence of lung cancer in the LDCT arm of the NLST trial^3^. The NLST trial population aligns with the criteria set by the European Commission recommendations used in the selection of cost‐related studies for this review. All costs identified in the included studies were first adjusted for inflation using the Gross Domestic Product (GDP) deflator index [24] and then converted to euros using the European Central Bank's exchange rates for July 2022.

Since this systematic review was carried out in the context of the update of the European Code Against Cancer project and corresponding methodology [15], we rated the confidence in the evidence for each outcome following the methodology implemented by the National Institute of Environmental Health Sciences (United States NIESH), adapted from the GRADE methodology [25]. We developed a GRADE evidence profile, summarizing the evidence for each outcome result, the relative and absolute effects of the intervention, and certainty of evidence.

## 3 Results

### 3.1 Search results

**Fig. 1:**
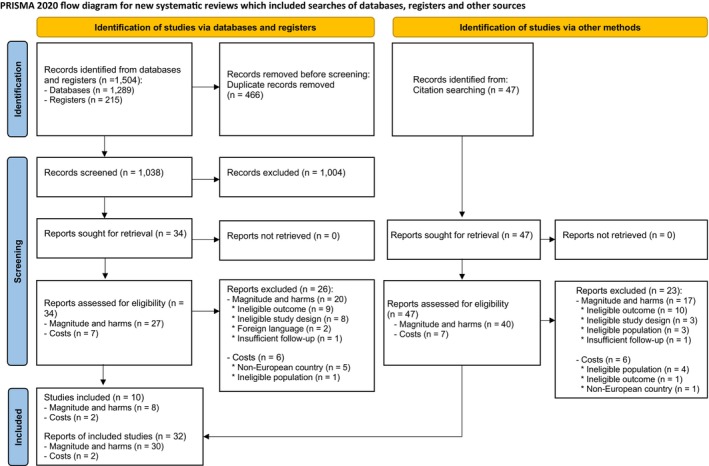
PRISMA flow diagram.

Our search strategy across databases and registers yielded 1504 records. After removing duplicates, we screened 1038 titles and abstracts, excluding 1004 of them. This left 34 potentially eligible records. After full‐text assessment, we excluded 26 records and included 8. We also identified 47 additional records through citation searching, of which we included 24. In total, we included 10 studies (eight RCTs and two cost‐related studies), reported across 32 publications, in our review (8 from database searches and 24 from backwards citation searches) (see Fig. 1). We report the reasons for exclusion in Table S2.

### 3.2 Characteristics of included studies

We included eight randomized trials and two cost‐related studies, reported in 32 publications [3, 9, 10, 24, 25, 26, 27, 28, 29, 30, 31, 32, 33, 34, 35, 36, 37, 38, 39, 40, 41, 42, 43, 44, 45, 46, 47, 48, 49, 50, 51, 52, 53, 54]. Four of these trials (ITALUNG [42], MILD [45], UKLS [31], NLST [3]) provided information for the evaluation of the magnitude of LDCT‐related overdiagnosis. DANTE [55], DLCST [48], LUSI [30] and NELSON [51] did not have the required follow‐up period of 6 years to be included in the overdiagnosis analysis. Seven of the eight included studies provided data on overdiagnosis‐related harms (DANTE [37], DLCST [48], ITALUNG [39], LUSI [30], NELSON [51], UKLS [31], NLST [3]). One study (MILD) did not report on the harm‐related outcomes of interest to this review (Table 1). Costs associated with overdiagnosis were assessed based on information reported in two studies [42, 47] (Table 2).

**Table 1:** Characteristics of included studies evaluating the magnitude and harms associated with overdiagnosis of LDCT. BAC, bronchioloalveolar carcinoma; CXR, chest x‐ray; DANTE, the detection and screening of early lung cancer by Novel imaging Technology trial; DLCST, the Danish lung cancer screening trial; FEV1, forced expiratory volume in 1 second; ITALUNG, the Italian lung cancer screening trial; kV, kilovolts; kvp, kilovoltage peak; LDCT, low‐dose computed tomography; LUSI, the Lung Cancer Screening Intervention trial; mAs, milliamperes; MILD, the Multicentric Italian lung detection trial; NELSON, Nederlands‐Leuvens Longkanker Screenings Onderzoek trial; NLST, the National lung screening trial; UKLS, the UK Lung Cancer Screening trial.

Study ID/country (*N* of sites)	Population			Intervention features			
	Eligibility criteria (including age and smoking status)	*N*	Sex distribution	LDCT type and parameters	Comparator	Frequency of scanning and duration or *n* of rounds	Follow‐up (after the last screening round)
Low‐dose computed tomography versus no screening							
DANTE/Italy(3)	Male, aged 60–74, smokers ≥ 20 pack years (current or quit < 10 years prior to actual)	LDCT: 1264; Control: 1,186	100% male	Size/140 kvp, 40 mA	No screening	Annual/Duration 5y	Unclear^a^
DLCST/Denmark(1)	Aged 50–70, asymptomatic, current or former smokers (former smokers had to have quit after age of 50 and < 10 years ago) with ≥ 20 pack‐year history of smoking, able to climb 2 flights of stairs (36 steps) without pausing FEV1 ≥ 30% predicted	LDCT: 2052 No screening: 2052	LDCT: (1147 males, 905 females); No screening: (1120 males, 932 females)	Size and volumetric/120 kV, 40 mAs	No screening	Annual/Duration 5 y	5y after last screening
ITALUNG/Italy (3)	Aged 55–69, resident in the trial catchment area, current smoker or former smoker (quit < 10 years) with at least a 20 pack‐year history	LDCT: 1613; Control: 1593	LDCT arm (1035 males, 578 females); control arm (1039 males, 554 females)	Size/120 kVP to 140 kVP, 20 mA to 43 mA	No screening	Annual/Duration 4y	Median 11 y
LUSI/Germany(1)	Age 50–69 years old with a smoking history of at least 40 pack years. If under the age of 60, current smokers or ceased smoking within the last 5 years	LDCT: 2029; control: 2023	LDCT (1315 males, 714 females); control (1307 males, 716 females)	Size and Volumetric/1.6 mSV to 2 mSV radiation exposure reported per scan	No screening	Annual/Duration 5 y	Median 5.73y after last screening
MILD/Italy(1)	Current or former smokers aged ≥ 49 years (having quit within 10 years of recruitment) with at least 20 pack years of smoking, and no history of cancer within previous 5 years	Biennial: 1186; Annual: 1190; Control: 1723	biennial LDCT arm (813 males, 373 females); annual LDCT arm (814 males, 376 females); control arm (1090 males, 633 females)	Volumetric/120 kV, 30 mAs	No screening	Biennial and annual arms/Duration of screening: 10 years (median 4 scans in biennial arm, 7 scans in annual arm)	Median 6y after last screening
NELSON/The Netherlands and Belgium(4)	Aged 50–75, smoked > 15 cigarettes/day during > 25 years or smoked > 10 cigarettes/day over > 30 years; current or former smokers who quit smoking ≤ 10 years ago	LDCT: 7900; control: 7892	LDCT arm (6583 males, 1317 females); control arm (6612 males, 1277 females, 3 missing)	volumetric/30 mA and 120 kVP to 140 kVP	No screening	Baseline, year 1, year 3 and year 5.5/Duration: 5.5 y	5.5. years after last screening
UKLS/England(2)	Aged 50–75 at high risk based on risk criteria of the Liverpool Lung Project (LLP) risk‐prediction model (based on age, sex, smoking history and other risk factors)	LDCT: 2028; Control: 2027	LDCT arm (1529 males, 499 females); control arm (1507 males, 520 females)	size and volumetric/90 kVp to 140 kVp, mA setting adjusted to achieve volume CT dose index	No screening	Annual/Duration 1 y	Median 6 y after last screening
Low‐dose computed tomography versus x‐ray							
NLST/USA(33)	Aged 55–74, with a ≥ 30 pack years of cigarette smoking history, current or former smokers (quit smoking within the previous 15 years)	LDCT: 26 722, CXR: 26 732	LDCT arm (male 15 770, female 10 953); CXR arm (male 15 763, female 10 970)	Size/120 kVp to 140 kVp and 20 mAs to 60 mAs	CXR	Baseline scan, then at 1 year, 2 years, and 2.5‐year intervals/Duration 3 y	Median 8.3 after last screening

a Recruitment occurred from 2001 to 2006; subjects underwent 5 rounds of screening. Follow‐up continued until 2012;

**Table 2:** Characteristics of included studies evaluating the costs associated with overdiagnosis. ID, Identification; LDCT, Low‐dose computed tomography.

Study ID	Country	Strategy	Periodicity	Adherence	Time horizon	Perspective	Modeling approach	Included costs	Currency of cost and year	Sensitivity analysis	Source of funding
Pan 2024	United Kingdom	LDCT (volumetri based screening)	Annual	100%	Lifetime	Societal	Decision tree and Markov	Direct and indirect medical costs	Pound (2020)	Discount, screening and treatment costs, utilities	AstraZeneca
Treskova 2017	Germany	LDCT (volumetri based screening)	Annual	100%	Lifetime	Health insurance	Microsimulation	Direct medical costs	Euro, year NR (published in 2017)	LDCT sensitivity, adherence, screening and treatment costs and periods	German Federal Ministry of Education and Research

### 3.3 Risk of bias in included studies

We assessed the risk of bias in studies estimating the magnitude of overdiagnosis, overdiagnosis‐related harms, and associated costs. Four studies evaluating overdiagnosis estimates raised high or some concerns about the risk of bias, and all studies on overdiagnosis‐related harms were deemed at low overall risk of bias. Similarly, among the two studies assessing overdiagnosis‐related costs, one lacked sufficient detail on model validation and internal verification. Detailed methodological limitations are available in Figs S1–S3.

### 3.4 Results of the included studies

Details on overdiagnosis, participation, and contamination in included trials are reported in Table S3.

#### 3.4.1 Comparison: LDCT Versus no screening

**Fig. 2:**
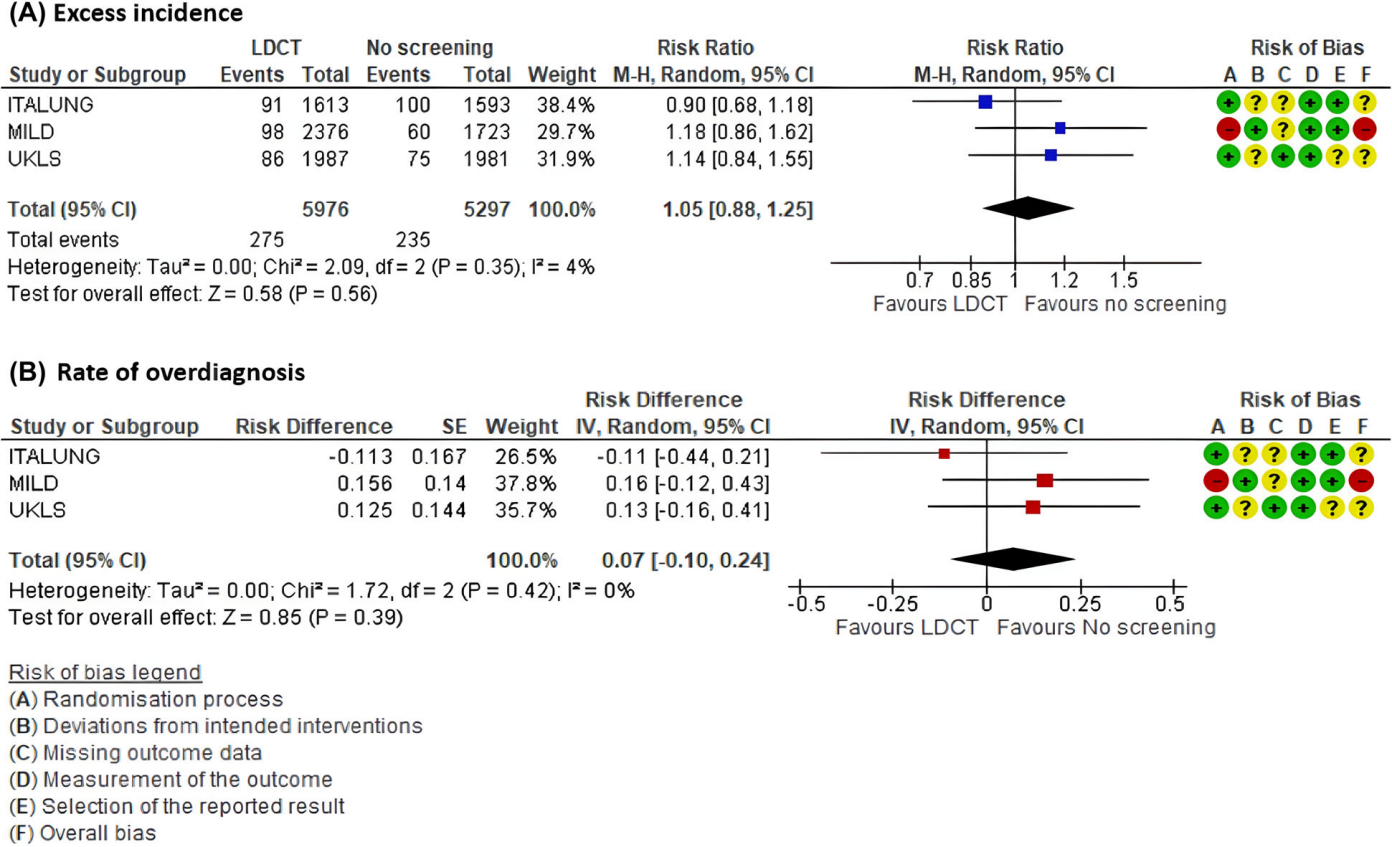
Magnitude of overdiagnosis. Comparison: LDCT versus no screening. (A) Excess of incidence (public perspective). (B) Rate of overdiagnosis (clinical perspective).

Three RCTs (ITALUNG [42], MILD [46], UKLS [33]) provided enough information for the calculation of overdiagnosis magnitude with a follow‐up of at least 6 years after the last screening round for comparing LDCT versus no screening. ITALUNG had a median follow‐up after screening of 11 years, and MILD and UKLS had a median follow‐up of six years. From the public health perspective, we found an excess incidence RR 1.05 (95% confidence interval [CI] 0.88 to 1.25) (Fig. 2A); see details of individual studies' results on overdiagnosis, participation, and contamination in included trials in the Table S3.

The rate of overdiagnosis from the clinical perspective was 0.07 (95% CI −0.10 to 0.24); 3 trials (ITALUNG [42], MILD [46], UKLS [33]); 11 273 participants (Fig. 2B).

Harms were reported in seven trials (DANTE [37], DLCST [10], ITALUNG [42], LUSI [30], NELSON [32], UKLS [33], and NLST [9]). Three studies (DANTE, DLCST, and ITALUNG) evaluated complications due to treatment in LDCT and reported the number of deaths after lung cancer treatment, and one (LUSI) reported the number of biopsies (Table 3).

**Table 3:** Complications and biopsies in LDCT‐related overdiagnosis for the comparison: LDCT versus no screening. DANTE, the Detection and screening of early lung cancer by Novel imaging Technology trial; DLCST, the Danish Lung Cancer Screening trial; E, events; ITALUNG, the Italian Lung Cancer Screening trial; LDCT, Low‐dose computed tomography; LUSI, the Lung Cancer Screening Intervention trial; *N*, number of participants.

Study ID	Outcome description	Harmful events in LDCT participants (E/*N*, %)	Lung cancer incidence in the LDCT arm^b^ (E/*N*; %)	Overdiagnosed participants in the LDCT arm (E/*N*)^a^, ^c^	Harmful events in overdiagnosed LDCT participants^d^ (%)	Absolute risk in LDCT overdiagnosed participants
Complications due to treatment						
DANTE	Deaths after surgical treatment	3/90; (3.33%)	104/1264 (8.23%)	5/1264 (0.4%)	0.013%	13 more deaths after surgery per 100 000 LDCT participants
DLCST	Deaths due to surgical treatment	1/11; (9.09%)	17/2052 (0.82%)	1/2052 (0.04%)	0.004%	4 more deaths after surgery per 100 000 LDCT participants
ITALUNG	Deaths after surgical treatment	2/67; (2.98%)	67/1613 (4.15%)	3/1613 (0.21%)	0.006%	6 more deaths after surgery per 100 000 LDCT participants
Biopsy						
LUSI	Number of biopsies	67/1881; (3.6%)	69/1881^e^ (3.7%)	3/1881 (0.18%)	0.17%^f^	170 more biopsies per 100 000 LDCT participants

a We used the overdiagnosis rate estimated through our meta‐analysis.

b Calculated with *N* of lung cancers / *N* of participants in the LDCT arm.

c Calculated based on the overdiagnosis estimate of this review (5%) * lung cancer incidence rate.

d Calculated with harmful events rate * % of overdiagnosis participants in the LDCT arm.

e 67/69 (97% of lung cancer cases underwent biopsies biopsy rate in lung cancer cases).

f Biopsy rate in lung cancer cases * % of overdiagnosis participants in the LDCT arm.

Two trials (NELSON [32], UKLS [33]) reported psychological effects such as depression and quality of life for this comparison (Table 4).

**Table 4:** Psychological harms: Quality of life and depression for the comparison: LDCT versus no screening. Mental component summary of short‐form 12 (SF‐12) and EuroQol questionnaire visual analogue scale (EQ‐5D VAS): lower scores indicate worse quality of life. Hospital Depression Scale: higher scores indicate worse outcome. CI, confidence interval; EQ5D, Euroqol 5 dimension; NELSON, Nederlands‐Leuvens Longkanker Screenings Onderzoek trial; SD, standard deviation; SF, short‐form; UKLS, the UK Lung Cancer Screening trial; VAS, visual analog scale.

Study ID	Outcome		*N*	Baseline	*N*	T2	Change (baseline‐T2)
NELSON		Time point of evaluation				At 2 years	Baseline to 2 years
	Quality of life (true +) Mental component summary of short‐form 12 (SF‐12)	LDCT	658		609		
		Mean ± SD		51.66 ± 11.69		52.50 ± 11.31	−0.84 (−2.11 to 0.43)
		No screening	630		322		
		Mean ± SD		51.72 ± 13.17		51.69 ± 10.49	0.03 (−1.51 to 1.57)
	Between‐group difference	Mean (95% CI)				0.81 (−0.65 to 2.27)	**−0.87 (−2.86 to 1.12)**
NELSON		Time point of evaluation				At 2 years	Baseline to 2 years
	Quality of life (true +) EuriQol questionnaire visual analogue scale (EQ‐5D VAS)	LDCT	658		609		
		Mean ± SD		79.19 ± 15.28		79.53 ± 14.8	−0.34 (−2.00 to 1.32)
		No screening	630		320		
		Mean ± SD		78.50 ± 17.26		77.45 ± 13.71	1.05 (−0.97 to 3.07)
	Between‐group difference	Mean (95% CI)				2.08 (0.17 to 3.99)	**−1.39 (−4.00 to 1.22)**
UKLS		Time point of evaluation				At 2 years	Baseline to 2 years
	Depression Hospital depression scale	LDCT	2018		2018		
		Mean ± SD		2.53 ± 2.29		2.77 ± 2.75	−0.24 (−0.40 to −0.08)
		No screening	2019		2019		
		Mean ± SD		2.81 ± 2.52		3.01 ± 2.98	−0.20 (−0.37 to 0.03)
	Between‐group difference	Mean (95% CI)				−0.24 (−0.42 to −0.06))	**−0.04 (−0.27 to 0.19)**

When 95% confidence intervals of between‐group differences do not cross 0, results can be considered statistically significant.

For the quality of life in true positives, results suggest that by 2 years, there may be little to no difference in the mental component of the quality of life (MD −0.87; 95% CI: −2.86 to 1.12) but that there may be a decrease in the overall quality of life assessed with EuroQoL (MD −1.39; 95% CI: −4.00 to 1.22) in LDCT participants compared to no screening. Regarding depression with the Hospital Depression Scale, results suggest there may be little to no difference in the depression score for LDCT participants compared to no screening (MD −0.04; 95% CI: −0.27 to 0.19).

**Fig. 3:**
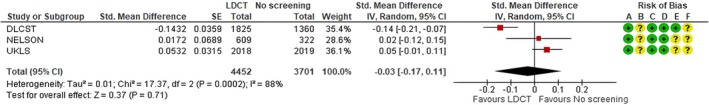
Overdiagnosis‐related harms: Anxiety. Comparison: LDCT versus no screening.

We pooled data from three studies (DLCST [48], NELSON [51] and UKLS [31]) reporting anxiety scores in LDCT screened patients and found that LDCT may result in little to no differences in anxiety score (MD −0.03; 95% CI: −0.17 to 0.11) (Fig. 3).

Only one RCT (ITALUNG) provided data on cumulative radiation dose over four years. In ITALUNG, during the 4 years of follow‐up, the cumulative effective dose of radiation was 3.35 Sv per 1000 subjects (0.83 mSv per subject per y) using the LDCT scanner (low‐dose 4‐mm collimation, yielding four 1‐mm‐thick sections).

### 3.5 Costs associated with overdiagnosis

Two studies provided information on LDCT screening‐related costs for the population of interest to this review. The two studies providing information on LDCT screening‐related costs were conducted, accounting for costs from the UK and Germany, respectively. While one of them evaluated the costs of LDCT screening from a societal perspective (i.e., including both direct and indirect costs) [21], the other used the provider's perspective (including only direct costs). Their results are summarized below (Table 5), and the certainty in this evidence is in Table 6.

**Table 5:** Results of cost‐related included studies. LDCT, Low‐dose computed Tomography; NI, not included; NR, Not reported.

Study ID	Strategy	Age group	Country	Direct costs of LDCT screening per person (€, 2022^a^)	Direct costs with treatment per person (€, 2022^a^)	Indirect costs of LDCT screening per person (€, 2022^a^)	Indirect costs with treatment per person (€, 2022^a^)	Discount (%)	Total costs of screening per person (discounted) (€, 2022^a^)	Total costs of no screening per person (discounted) (€, 2022^a^)	Difference in costs of screening (LDCT versus no screening) per person (discounted) (€, 2022^a^)	Overdiagnosis estimate in the present review	Lung cancer incidence	N of overdiagnosed people per 100,000 LDCT participants	Additional costs of overdiagnosis
Pan 2024	LDCT (volumetric based)	55–75	United Kingdom	€84.85^b^	NR	NR^c^	NR^c^	3.5%	€23 056.27^d^ ^–^ ^f^	€16 301.53^d^ ^–^ ^f^	€6754.74^d^, ^e^	5%	6.37%	300	€2,026,422.00^e^ per 100 000 participants
Treskova 2017	LDCT (volumetric based)	55–74	Germany	€172.65^g^	NR	NI	NI	3%	€4678.31^f^, ^g^, ^h^	€3469.61^f^, ^g^, ^h^	€1208.70^g^	5%	6.37%	300	€362,610.00^h^ per 100 000 participants

a Monetary value adjusted by inflation using gross domestic product (GDP) deflator index.

b Considering only the costs of LDCT. Costs of screening were not reported.

c Indirect costs include productivity loss, informal care, and transportation costs, in addition to the direct healthcare costs from a healthcare system perspective. However, the inputs for indirect costs were not reported.

d Converted to Euros using European Central Bank exchange rates for July 2022.

e Cost included recruitment, screening, diagnosis and treatment. The study included indirect costs related to productivity loss, informal care, and transportation costs, in addition to the direct healthcare costs from a healthcare system perspective. However, the inputs for indirect costs were not reported.

f We used the incidence of lung cancer in the LDCT arm of the NLST trial.

g The year was not reported in the study. We used the publication year for calculation.

h Costs included LDCT exams, staging tests, and lifetime treatment.

**Table 6:** Summary of findings for LDCT versus no screening. NA, not applicable.

Outcomes (design)	No of participants (number of studies)	Initial certainty of the evidence	Risk without LDCT	Relative effects	Anticipated absolute effects	Updated certainty of evidence
Overdiagnosis (Public health perspective)	11 273 (3)	High	4.4%	RR 1.05 (0.88 to 1.25)	222 more overdiagnosed cases per 100 000 participants (from 532 fewer to 1109 more)	⊕⊕○○^a^, ^b^ Low
Overdiagnosis (Clinical perspective)	11 273 (3)	High	NA	0.07 (−0.10 to 0.24)	7000 more lung cancers overdiagnosed per 100 000 lung cancers detected (10 000 less to 24 000 more)	⊕⊕○○^a^, ^b^ Low
Overdiagnosis‐related harms (Complications due to treatment: Deaths after surgical treatment)^c^	5656 (2)	High	NA	NA	6 more per 100 000 participants (from 4 more to 13 more)	⊕⊕○○^b^, ^d^ Low
Overdiagnosis‐related costs: Direct and indirect costs of overdiagnosis (societal perspective): additional costs of overdiagnosis	Cost‐effectiveness (1)	High	NA	NA	€2,026,422.00 per 100 000 participants	⊕⊕○○ Low^e^, ^f^
Overdiagnosis‐related costs: Direct costs of overdiagnosis (healthcare care provider's perspective): additional costs of overdiagnosis	Cost‐effectiveness (1)	High	NA	NA	€362,610.00 per 100 000 participants	⊕○○○ Very low^g^, ^h^

a We downgraded the certainty of evidence by one level due to serious methodological limitations in the included studies (high risk of bias on the randomization process and some concerns about the risk of bias due to deviations from intended interventions, missing outcome data, and the selection of the reported results).

b We downgraded the certainty of evidence by one level due to serious imprecision.

c One of the RCTs reported the death within 60 days after surgical treatment, while the other RCTS did not provide follow‐up time.

d We downgraded the certainty of evidence by one level due to serious methodological limitations (some concerns on the risk of bias in the randomization process, deviations from intended interventions, and missing outcome data).

e We downgraded the certainty of evidence by one level due to risk of bias regarding serious methodological limitations (there was no clear validation of the model).

f Includes the cost of recruitment, screening, diagnosis, and treatment (direct and indirect). We downgraded the certainty of evidence by one level due to indirectness because the lung cancer incidence was taken from the LDCT arm of the NLST trial and the overdiagnosis estimate from the present review.

g Includes only costs of diagnostic workup (CT supported biopsy, histology, head MRI, contrast medium, pneumothorax) (no indirect costs accounted for). We downgraded the certainty of evidence by two levels due to indirectness, as the costs of lung cancer treatment tend to be higher in Germany than costs in other European countries. Additionally, the lung cancer incidence was taken from the LDCT arm of the NLST trial and the overdiagnosis estimate from the present review.

h We downgraded the certainty of evidence by one level due to imprecision, as the sensitivity analysis shows that screening‐related costs, especially cost per CT exam across countries, may lead to important variability in costs estimate.

We evaluated the certainty of evidence and reported a summary of findings for the comparison LDCT versus no screening (Table 6).

#### 3.5.1 Comparison: LDCT Versus chest x‐ray

**Fig. 4:**
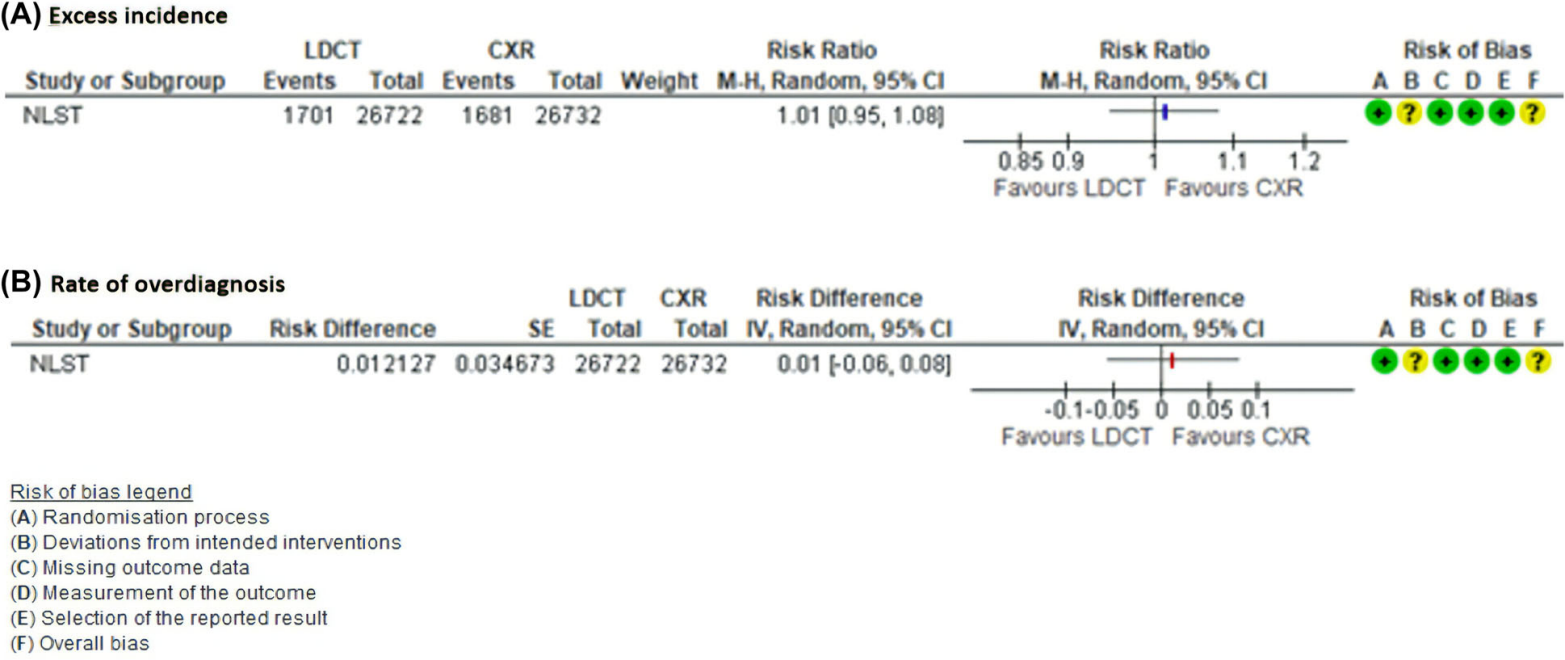
Magnitude of overdiagnosis for the comparison: LDCT versus CXR. (A) Excess of incidence (public perspective). (B) Rate of overdiagnosis (clinical perspective).

NLST was the only study to provide data on the incidence of lung cancer with a follow‐up of at least 6 years. It had a median follow‐up of 8.3 years after screening. For the LDCT versus CXR, we found a rate of overdiagnosis from the public perspective RR 1.01 (95% CI 0.95 to 1.08); 1 trial; 53 454 participants (Fig. 4A). For the LDCT versus CXR, we found a rate of overdiagnosis from the clinical perspective 0.01 (95% CI −0.06 to 8); 1 trial; 53 454 participants (Fig. 4B). There were no studies identified that addressed the outcomes: repeat computed tomography (CT) or high radiation dose imaging. Also, we did not find studies comparing costs associated with LDCT versus chest x‐ray.

The NLST trial reported psychological harms such as anxiety and quality of life (Table 7). Regarding quality of life, for participants with true positive results, the crude between‐group comparison at 6 months showed a mean difference (MD) of −0.87 (95% CI: −2.86 to 1.12). However, when adjusting for multiple confounders, the analysis suggests that there may be a decrease in the mental component of quality of life, with a MD of −4.15 (95% CI: −6.27 to −2.03). Adjustments were made for site of origin, baseline score, days since baseline evaluation, baseline age, sex, years of education, marital status, smoking status, race, ethnicity, number of prior suspicious for lung cancer screens, number of prior significant incidental findings (SIFs) in screens, and the statistical interaction between days since baseline and baseline score, and for 1‐month parameters, whether participants knew their screening results at the time of questionnaire completion.

**Table 7:** Psychological harms for low‐dose computed tomography versus chest x‐ray. Mental component of Spielberger State Trait Anxiety Inventory (STAI Form Y‐1): higher scores indicate worse anxiety. Mental component summary of short‐form 12 (SF‐12): lower scores indicate worse quality of life. CI, confidence interval; NLST, the National Lung Screening Trial; SD, standard deviation; T2, timepoint 2.

Study ID	Outcome		*N*	Baseline	*N*	T2	Change (Baseline‐T2)
NLST		Time point of evaluation				At 6 months	Baseline to 6 months
	Anxiety (true +) Mental component o Spielberger State Trait Anxiety Inventory (STAI Form Y‐1)	LDCT	1947		1947		
		Mean ± SD		41.06 ± 15.10		37.69 ± 12.04	−3.37 (−4.23 to −2.51)
		Chest x‐ray	865		865		
		Mean ± SD		39.43 ±11.66		39.38 ±14.47	−0.05 (−1.29 to 1.19)
	Between‐group difference (unadjusted)	Mean (95% CI)				−1.69 (−2.79 to −0.59)	−3.32 (−1.81 to −4.83)
	Adjusted analysis						**1.38 (1.05 to 1.82)** *
NLST		Time point of evaluation				At 6 months	Baseline to 6 months
	Quality of life (true +) Mental component summary of short‐form 36 (SF‐36) at baseline	LDCT	1947		1947	
		Mean ± SD		52.03 ± 11.04		46.30 ± 13.65	−5.73 (−6.51 to −4.95)
		Chest x‐ray	865		865	
		Mean ± SD		53.77 ± 8.57)		46.22 ± 12.17	−7.55 (−8.54 to −6.56)
	Between‐group difference (unadjusted)	Mean (95% CI)				0.08 (‐0.93 to 1.09)	1.82 (0.56 to 3.08)
	Adjusted analysis						**−4.15 (−6.27 to −2.03)** *

* Adjusted for site of origin, baseline score, days since baseline evaluation, baseline age, sex, years of education, marital status, smoking status, race, ethnicity, number of prior suspicious for lung cancer screens, number of prior significant incidental finding in screens and the statistical interaction between days since baseline and baseline score, and for 1‐month parameters, whether participants knew results of index screen prior to 1 month HRQoL.

When 95% confidence intervals of between‐group differences do not cross 0, results can be considered statistically significant.

Regarding anxiety, for participants with true positive results, the crude between‐group comparison at 6 months showed a MD of −3.32 (95% CI: −4.83 to −1.81). However, when adjusting for multiple confounders, the analysis suggests that there may be an increase in anxiety scores, with a relative risk (RR) of 1.38 (95% CI: 1.05 to 1.82). The same set of confounders used in the quality‐of‐life analysis was adjusted for in this analysis.

We evaluated the certainty of evidence and reported a summary of findings for the comparison LDCT versus CXR (Table 8).

**Table 8:** Summary of findings for LDCT versus chest x‐ray. NA, not applicable.

Outcomes (design)	Participants (studies)	Initial certainty of the evidence	Risk without LDCT	Relative effects	Anticipated absolute effects	Updated certainty of evidence
Overdiagnosis (Public health perspective)	53 454 (1)	High	6.3%^a^	RR 1.01 (0.95 to 1.08)	63 more overdiagnosed cases per 100,000 participants (from 314 fewer to 503 more)	⊕⊕⊕○^b^ Moderate
Overdiagnosis (Clinical perspective)	53 454 (1)	High	NA	0.01 (−0.06 to 0.08)	1000 more lung cancers overdiagnosed per 100 000 lung cancers detected (6000 fewer to 8000 more)	⊕⊕⊕○^a^ Moderate

a Risk without LDCT was calculated using the event rate in the non‐screening groups.

b We downgraded the certainty of evidence by one level due to serious methodological limitations (some concerns about the risk of bias due to deviations from intended interventions).

## 4 Discussion

### 4.1 Main findings

This is the first systematic review to evaluate overdiagnosis‐related harms of lung cancer screening. Our findings suggest that LDCT screening may increase lung cancer overdiagnosis, compared to no screening and likely slightly increases overdiagnosis compared to CXR, with an additional societal cost, but does not appear to substantially increase overtreatment‐related harms. LDCT screening in high‐risk populations may lead to a slight increase in overdiagnosis compared to no screening, corresponding to approximately 222 additional accumulated cases per 100 000 participants screened. Similarly, when compared to chest X‐ray (CXR), LDCT also showed a small increase in overdiagnosis risk, corresponding to approximately 63 additional cases per 100 000 participants screened. Bonney 2022 [56] found that the absolute increase in overdiagnosis for screening compared to usual care was 18 000 per 100 000 detected cases. In contrast, Brodersen et al. [4] reported a somewhat higher estimate, with an absolute overdiagnosis of 38 000 per 100 000 detected cases.

### 4.2 Results in the context of previous studies

A key factor contributing to discrepancies across studies is variation in follow‐up duration. To minimize the inflation of overdiagnosis estimates due to lead‐time bias, our review included data from trials with follow‐up periods exceeding six years. In comparison, Bonney et al. employed longer follow‐up periods (> 10 years), while Brodersen et al. relied on studies with shorter follow‐up (3–5 years). Our choice of a ≥ 6‐year cut‐off represents a compromise between these approaches, supported by modeling work suggesting that the sojourn time of some potentially lethal tumor types may extend up to this period [19, 20]. As overdiagnosis is time‐dependent, including trials with shorter follow‐up durations, such as the DLCST, may overestimate rates by misclassifying indolent tumors as clinically significant. This effect was demonstrated in the extended analysis of the National Lung Screening Trial [54], where longer follow‐up helped clarify the clinical trajectory of screen‐detected cancers. Li et al. [55] also observed a 3–4% annual decline in excess incidence during post‐screening follow‐up, particularly among older individuals and across specific histological subtypes of lung cancer.

Beyond follow‐up length, heterogeneity in trial design and definitions of overdiagnosis further complicates interpretation. Reported overdiagnosis rates ranged from −4 to 67% across studies, largely due to differences in study populations, screening protocols, and diagnostic criteria [3, 10, 30, 33, 42]. Depending on how overdiagnosis is calculated, estimates can reflect either a public health or a clinical perspective. Some trials define overdiagnosis as the proportion of screen‐detected cancers that are considered indolent. This reflects a clinical viewpoint, focusing on the likelihood that a cancer found through screening would not have caused harm during the patient's lifetime. In contrast, other studies assess excess cancer incidence in the screened population compared to an unscreened group, which provides a population‐level (public health) perspective, capturing the broader impact of screening programs on disease burden. For instance, Patz et al. proposed two distinct metrics to capture these perspectives: (1) the probability that a screen‐detected lung cancer is overdiagnosed, which represents the clinical perspective, and (2) the number of overdiagnosed cases relative to the number of individuals needed to screen to prevent one lung cancer death, which reflects the public health perspective by weighing harms against population‐level benefits. To minimize lead‐time bias, we defined overdiagnosis as the persisting excess incidence of lung cancer after a minimum of six years of follow‐up without continued screening.

Structured nodule management systems, such as Lung‐RADS and the British Thoracic Society (BTS) guidelines, were developed to standardize follow‐up and minimize unnecessary investigations. By incorporating growth assessment, higher referral thresholds, and, specifically in the BTS guidelines, volumetry, these approaches aim to reduce false positives and overdiagnosis. Several trials included in our review (e.g., MILD, ITALUNG, UKLS) already employed volumetry‐based protocols, indicating that our estimates partly reflect contemporary practice, though they may still exceed what would be expected under strict adherence to these systems. Nevertheless, the findings remain highly relevant given the variability of real‐world implementation. In relation to harms associated with overdiagnosis, we observed considerable heterogeneity in how outcomes were defined and reported. For instance, the DANTE, DLCST, and ITALUNG trials assessed treatment‐related mortality in screened versus unscreened participants, but only DANTE and ITALUNG reported complete outcome data. This lack of consistency limits the comparability and synthesis of findings across studies. The psychological consequences of overdiagnosis were assessed in trials such as DLCST, NELSON, UKLS, and NLST using various measurement tools. However, all results were reported at the population level, precluding any estimation of the specific psychological burden attributable to overdiagnosis. This limitation highlights the need for standardized instruments capable of isolating and quantifying the emotional impact of overdiagnosed individuals.

Our study also explored the economic impact of lung cancer overdiagnosis. Even small increases in overdiagnosis rates can translate into economic burdens for health systems, primarily due to additional diagnostic tests, specialist consultations, and unnecessary treatments, some of which carry risks of complications and require long‐term follow‐up. As highlighted in Treskova 2017^49^, high detection and overdiagnosis rates can negatively affect the incremental cost‐effectiveness ratio (ICER), potentially leading to the inefficiency of LDCT screening by increasing treatment costs relative to screening costs. Moreover, when indirect costs are taken into account, the estimated economic burden of overdiagnosis rises substantially, from €362610.00 to €2026422.00 per 100 000 individuals screened.

### 4.3 Limitations and strengths

This study has some limitations, including inconsistent reporting of outcomes such as anxiety, quality‐of‐life impairments, and complications from unnecessary treatment, which limited our ability to quantify these effects. Additionally, the economic data varied widely in scope and methodology, restricting the generalizability of cost estimates across healthcare settings.

Nevertheless, this systematic review has several notable strengths. This work represents the most up‐to‐date systematic review of randomized evidence, including extended follow‐up. We conducted a comprehensive review including data from eight randomized trials (> 84 000 participants) and economic analyses. Previous reviews have often focused narrowly on incidence alone, without exploring overdiagnosis‐related harms and costs. By addressing overdiagnosis and its clinical, psychological, and economic consequences, we offer a multidimensional evidence base to inform decision‐making. Methodological rigor was ensured through Cochrane Rapid Review Methods and NICE guidelines, and long follow‐up periods minimized lead‐time bias. Our integration of diverse outcomes and use of GRADE to assess evidence certainty strengthen the relevance and transparency of the findings for both clinical and policy contexts.

### 4.4 Implications for practice and research

Although LDCT screening has demonstrated a reduction in lung cancer mortality, overdiagnosis remains a major concern, especially where different protocols for the management of pulmonary nodules are followed [57]. From a clinical perspective, healthcare professionals must follow the latest guidelines on the management of the findings on LDCT [58, 59, 60]. Imaging biomarkers are a useful way to limit overdiagnosis by assessing whether potential cancers are likely to be harmful prior to diagnosis. This means attention to balancing the benefits and harms of screening, ensuring that patients are adequately informed about the potential consequences of overdiagnosis. Additionally, from a health system perspective, resources allocated to treating indolent cancers could be redirected to other priorities.

Future research should prioritize strategies to mitigate the impact of overdiagnosis. This includes developing advanced algorithms to differentiate indolent from aggressive lesions, refining screening criteria, and conducting targeted studies using standardized psychological assessment tools. Efforts to quantify and reduce the unintended harms of screening will be essential to maximizing net benefit. Future studies should also address limitations using standardized reporting frameworks.

Our study has, by virtue of its broad perspective, integrating clinical, psychological, and economic outcomes, allowed conclusions that are informative to clinicians and policy‐makers alike. Overdiagnosis in lung cancer screening is likely to be a small contributor to harms, although further reduction through adherence to the latest guidelines is essential.

## 5 Conclusions

LDCT may result in higher rates of overdiagnosis compared to no screening. The differences between LDCT and chest X‐ray (CXR) screening are likely small. Overdiagnosis was associated with increased use of invasive procedures and treatment‐related harms, although such events may be relatively infrequent when considering only the overdiagnosed cases. From a societal perspective, the financial impact of overdiagnosis may vary depending on the availability of national healthcare resources. Notably, this review suggests that the magnitude of overdiagnosis associated with LDCT is lower than earlier estimates that considered shorter follow‐up periods. While some degree of overdiagnosis is unavoidable in lung cancer screening, improved risk‐based selection and adherence to guideline‐recommended nodule management may help reduce this burden to an acceptable level, particularly when weighed against the potential benefits of screening. The findings from this review were used to inform the decision‐making about including recommendations on lung cancer screening in the update of the European Code Against Cancer, 5th edition [61].

## Conflict of interest

CA works on the 4‐IN‐THE‐LUNG‐RUN study (EU grant) and the CanScreen project (EU grant), and is a member of the European Code Against Cancer working group (WHO‐IARC). Travel costs were covered for attending several meetings related to lung cancer screening. All other authors declare no conflict of interest.

## Authors contributions

AC, CE, CVA, DR, DRB, and MT conceived and designed the project. ACPNP and FKFS acquired and curated the data. ACPNP and FKFS analyzed and interpreted the data. AC, ACPNP, CCA, CE, CVA, DR, DRB, FKFS, IS, LTP, MMP, MT, and PAC carried out the investigation. ACPNP, CCA, CE, DR, FKFS, and PAC administered the project. ACPNP, FKFS, LTP, and MMP validated the findings. ACPNP and FKFS wrote the original draft. AC, ACPNP, CCA, CE, CVA, DR, DRB, FKFS, IS, LTP, MMP, MT, and PAC reviewed, edited, and approved the final version of the manuscript.

## IARC disclaimer

Where authors are identified as personnel of the International Agency for Research on Cancer/World Health Organization, the authors alone are responsible for the views expressed in this article and they do not necessarily represent the decisions, policy, or views of the International Agency for Research on Cancer/World Health Organization.

## Supporting information


**Fig. S1.** Risk of bias for magnitude of overdiagnosis estimates. Assessment of bias in the effect of assignment to intervention.

**Fig. S2.** Risk of bias for overdiagnosis‐related harm estimates. Assessment of bias in the effect of assignment to intervention.

**Fig. S3.** Risk of bias for costs associated with overdiagnosis estimates.

**Table S1.** Search strategy.

**Table S2.** Reasons for exclusion of studies.

**Table S3.** Overdiagnosis, participation, and contamination in included trials.

## Data Availability

This study did not generate new data. All data is available from the cited publications.
