# Supplementary figures and images for: Screening for lung cancer: A systematic review of overdiagnosis and its implications
Source: Mol Oncol. 2025 Nov 11;20(3):611–28. doi: 10.1002/1878-0261.70139 (PMC13042368; doi:10.1002/1878-0261.70139)

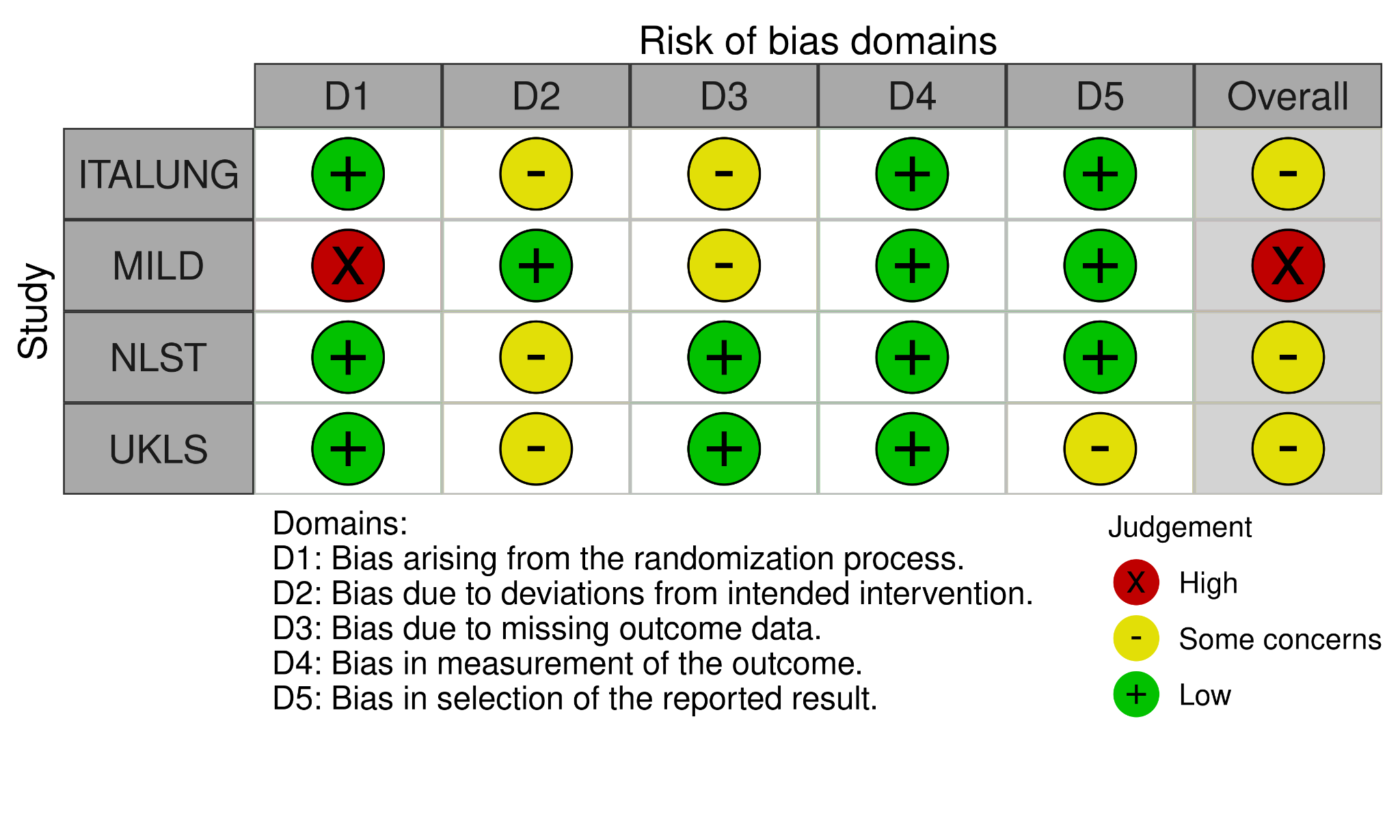

Supplement: Supplementary file 1 — Fig. S1. Risk of bias for magnitude of overdiagnosis estimates. Assessment of bias in the effect of assignment to intervention. [file MOL2-20-611-s004.tif]

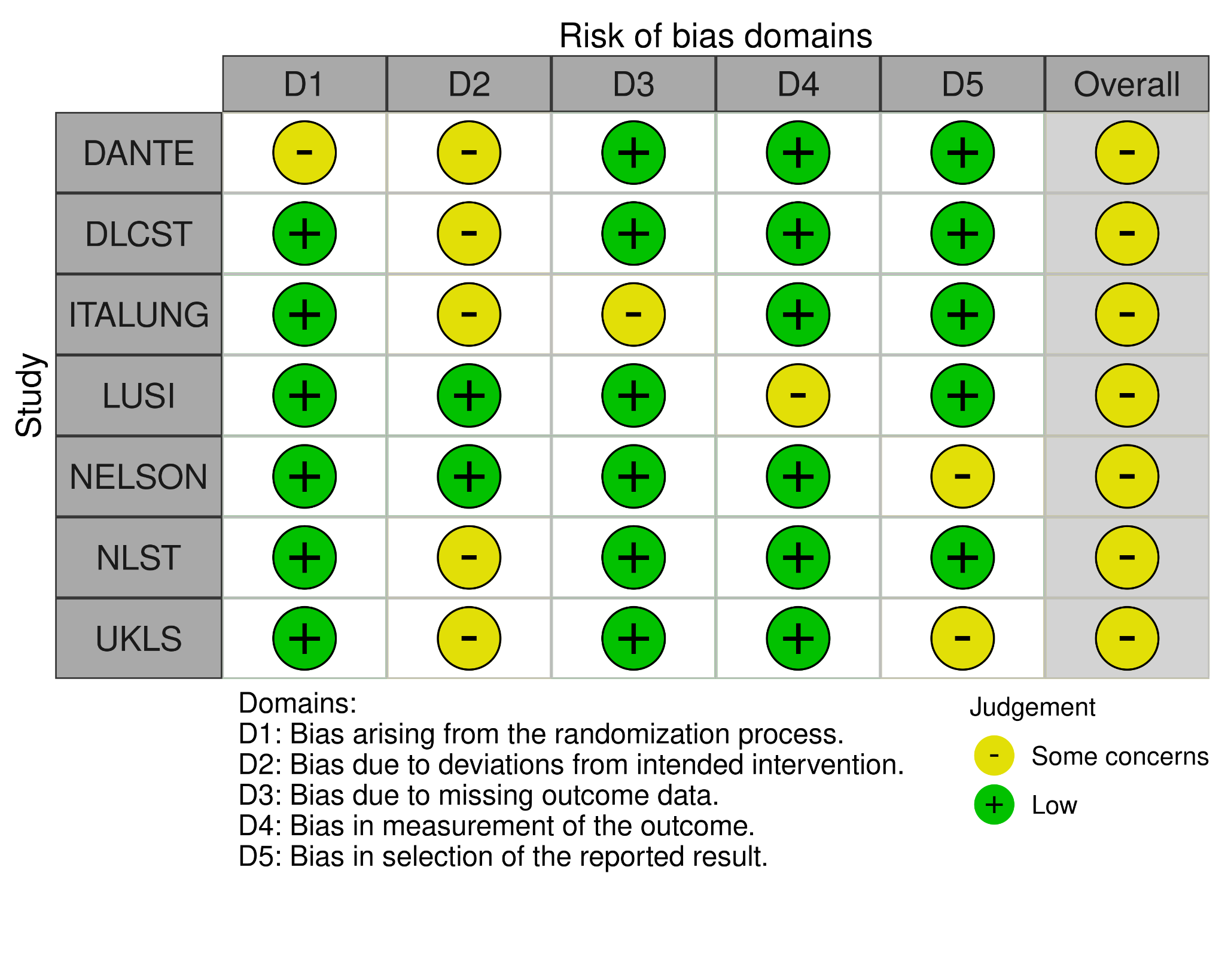

Supplement: Supplementary file 2 — Fig. S2. Risk of bias for overdiagnosis‐related harm estimates. Assessment of bias in the effect of assignment to intervention. [file MOL2-20-611-s003.tif]

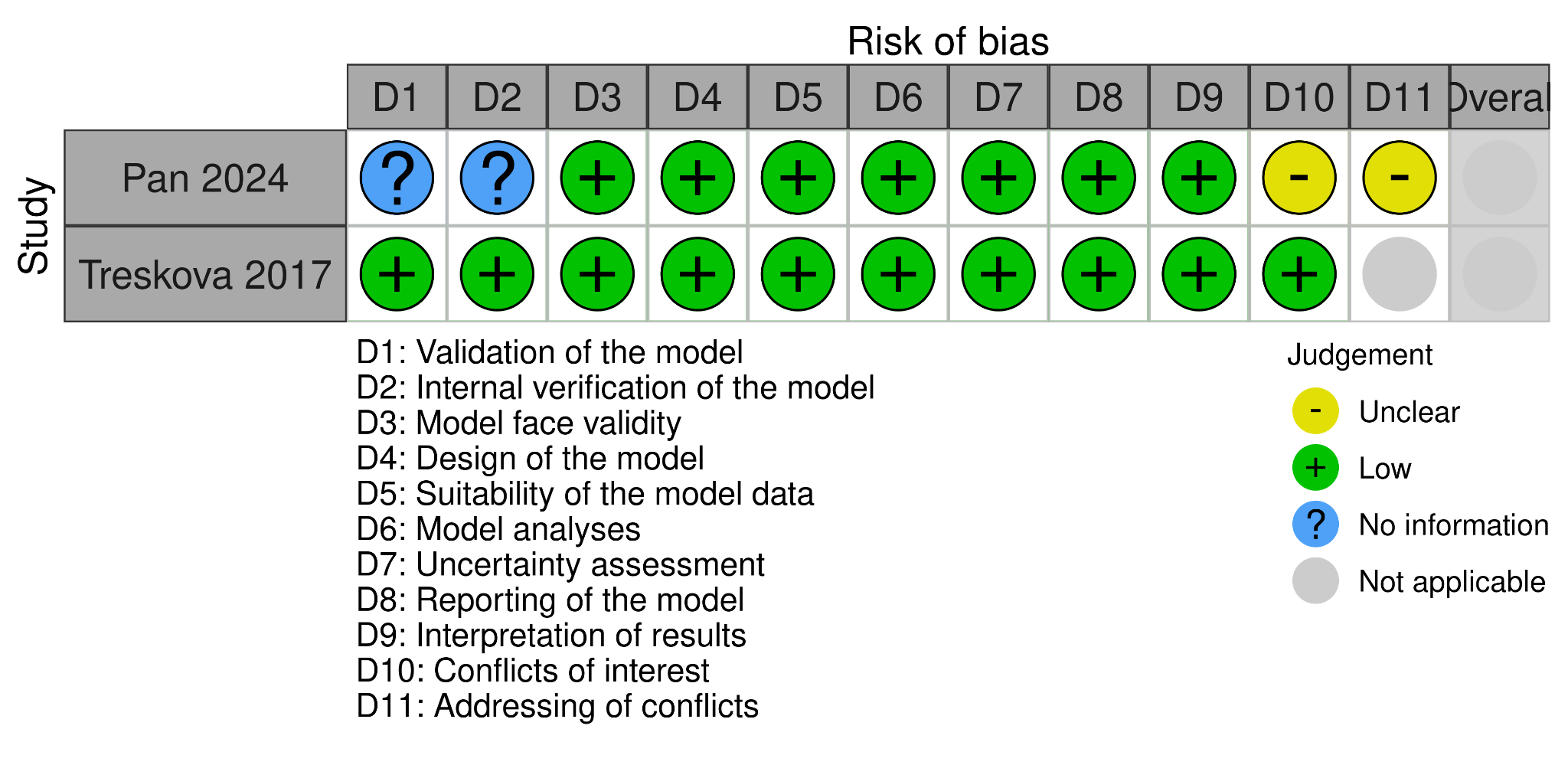

Supplement: Supplementary file 3 — Fig. S3. Risk of bias for costs associated with overdiagnosis estimates. [file MOL2-20-611-s002.tif]
