# Supplementary material for: Screening for lung cancer: A systematic review of overdiagnosis and its implications
Source: Mol Oncol. 2025 Nov 11;20(3):611–28. doi: 10.1002/1878-0261.70139 (PMC13042368; doi:10.1002/1878-0261.70139)
Supplement: Supplementary file 4 — Table S1. Search strategy. [file MOL2-20-611-s005.doc]

**Supplementary Table 1. Search strategy**

| **Database (search date)** | Search Strategy |
| --- | --- |
| **MEDLINE**  **PubMed**  **15/05/2024** | #1 "Lung Neoplasms"[Mesh] 284,45  #2 "Carcinoma, Squamous Cell"[Mesh] 154,431  #3 "lung cancer*"[tiab] 218,223  #4 "lung nodule*"[tiab] 5,396  #5 #1 OR #2 OR #3 OR #4 490,665  #6 "Early Detection of Cancer"[Mesh] 40,235  #7 "Mass Screening"[Mesh] 146,015  #8 screening[tiab] 716,048  #9 "early detection"[tiab] 90,147  #10 #6 OR #7 OR #8 OR #9 840,274  #11 "Medical Overuse"[Mesh] 15,336  #12 overdiagnos*[tiab] 6,73  #13 indolent[tiab] 15,428  #14 "excess number"[tiab] 422  #15 "excess cancer"[tiab:~3] 3,77  #16 "excess cancers"[tiab:~3] 607  #17 "excess incidence"[tiab:~3] 934  #18 #11 OR #12 OR #13 OR #14 OR #15 OR #16 OR #17 41,64  #19 #5 AND #10 AND #18 458 |
| **Cochrane Central Register of Controlled Trials**  **The Cochrane Library**  **Issue 4 of 12, April 2024**  **Date Run: 15/05/2024** | #1 MeSH descriptor: [Lung Neoplasms] explode all trees 12078  #2 MeSH descriptor: [Carcinoma, Squamous Cell] explode all trees 4415  #3 (lung NEXT cancer*):ti,ab 22725  #4 (lung NEXT nodule*):ti,ab 283  #5 #1 OR #2 OR #3 OR #4 29320  #6 MeSH descriptor: [Early Detection of Cancer] explode all trees 2550  #7 MeSH descriptor: [Mass Screening] explode all trees 5992  #8 screening:ti,ab 73098  #9 "early detection":ti,ab 3227  #10 #6 OR #7 OR #8 OR #9 76837  #11 MeSH descriptor: [Medical Overuse] explode all trees 682  #12 overdiagnos*:ti,ab 459  #13 indolent:ti,ab 1012  #14 (excess NEAR/3 (number OR cancer* OR incidence)):ti,ab 176  #15 #11 OR #12 OR #13 OR #14 2253  #16 #5 AND #10 AND #15 79 |
| **EMBASE**  **15/05/2024** | #1. 'lung cancer'/exp 510,422  #2. 'squamous cell carcinoma'/exp 236,904  #3. 'lung cancer*':ti,ab 326,623  #4. 'lung nodule*':ti,ab 9,266  #5. #1 OR #2 OR #3 OR #4 752,480  #6. 'mass screening'/exp 330,595  #7. 'early cancer diagnosis'/exp 15,091  #8. screening:ti,ab 993,052  #9. 'early detection':ti,ab 127,740  #10. #6 OR #7 OR #8 OR #9 1,251,161  #11. 'medical overuse'/exp 9,650  #12. overdiagnos*:ti,ab 9,589  #13. indolent:ti,ab 26,689  #14. (excess NEAR/3 (number OR cancer* OR incidence)):ti,ab 5,714  #15. #11 OR #12 OR #13 OR #14 49,852  #16. #5 AND #10 AND #15 792  #17. #16 AND [embase]/lim 752 |
