# Supplementary material for: Screening for lung cancer: A systematic review of overdiagnosis and its implications
Source: Mol Oncol. 2025 Nov 11;20(3):611–28. doi: 10.1002/1878-0261.70139 (PMC13042368; doi:10.1002/1878-0261.70139)
Supplement: Supplementary file 5 — Table S2. Reasons for exclusion of studies. [file MOL2-20-611-s001.doc]

**Supplementary Table 2. Reasons for exclusion of studies**

| **N** | **Author ID** | **Publications (Title)** | **Main reasons for exclusion** |
| --- | --- | --- | --- |
| **Magnitude and Harms of overdiagnosis** | | | |
| ***Identified through Database and registers search*** | | | |
| 1 | Aberle 2011 (NLST) | The National Lung Screening Trial: overview and study design | Ineligible outcome |
| 2 | Croswell 2010 (LSS) | Cumulative incidence of false-positive test results in lung cancer screening: a randomized trial | Ineligible outcome |
| 3 | Durham 2021 | Do competing causes of mortality contribute to overdiagnosis in lung cancer screening? | Ineligible study design |
| 4 | Gohagan 2004 (LSS) | Baseline findings of a randomized feasibility trial of lung cancer screening with spiral CT scan vs chest radiograph: the Lung Screening Study of the National Cancer Institute | Ineligible outcome |
| 5 | Goo 2022 | Potential Overdiagnosis with CT Lung Cancer Screening in Taiwanese Female: Status in South Korea | Ineligible study design |
| 6 | Haaf 2015 | Overdiagnosis in lung cancer screening: why modelling is essential. | Ineligible study design |
| 7 | Maldonado 2015 (NLST) | Noninvasive Computed Tomography-based Risk Stratification of Lung Adenocarcinomas in the National Lung Screening Trial. | Ineligible outcome |
| 8 | Meza 2024 | Assessing the impact of increasing lung screening eligibility by relaxing the maximum years-since-quit threshold: A simulation modeling study. | Ineligible study design |
| 9 | Meza 2021 | Evaluation of the Benefits and Harms of Lung Cancer Screening with Low-Dose Computed Tomography: Modeling Study for the US Preventive Services Task Force | Ineligible study design |
| 10 | NCT00006382 (LSS) | Lung Screening Study | Ineligible outcome |
| 11 | Pinsky 2020 | Long-term cancer risk associated with lung nodules observed on low-dose screening CT scans. | Ineligible outcome |
| 12 | Pinsky 2014 | Assessing the benefits and harms of low-dose computed tomography screening for lung cancer | Ineligible study design |
| 13 | Saghir 2011 (DLCST) | Contamination during 4 years of annual CT screening in the Danish Lung Cancer Screening Trial (DLCST) | Ineligible outcome |
| 14 | Saghir 2012 (DLCST) | CT screening for lung cancer brings forward early disease. The randomised Danish Lung Cancer Screening Trial: status after five annual screening rounds with low-dose CT | Insufficient follow-up |
| 15 | Seijo 2014 | Lung cancer screening with low-dose computed tomography | Ineligible study design |
| 16 | Silva 2018 | Long-Term Active Surveillance of Screening Detected Subsolid Nodules is a Safe Strategy to Reduce Overtreatment. | Ineligible study design |
| 17 | Stang 2016 | Early detection of lung cancer: Low-dose CT screening | Foreign language |
| 18 | Wang 2023 | The Introduction of Low-Dose CT Imaging and Lung Cancer Overdiagnosis in Chinese Women. | Ineligible outcome |
| 19 | Yip 2016 (NLST) | Lung Cancer Deaths in the National Lung Screening Trial Attributed to Nonsolid Nodules | Ineligible outcome |
| 20 | Zompatori 2012 (NLST) | Screening for lung tumor with low-dose spiral CT. Remarks upon the NLST trial | Foreign language |
| ***Identified through citation searching*** | | | |
| 1 | Aberle 2010 (NLST) | Baseline characteristics of participants in the randomized national lung screening trial | Ineligible outcome |
| 2 | Blanchon 2007 (DEPISCAN) | Baseline results of the Depiscan study: a French randomized pilot trial of lung cancer screening comparing low dose CT scan (LDCT) and chest X-ray (CXR) | Ineligible outcome |
| 3 | Dunn 2017 (UKLS) | The role of screening expectations in modifying short-term psychological responses to low-dose computed tomography lung cancer screening among high-risk individuals | Ineligible study design |
| 4 | Field 2016A (UKLS) | UK Lung Cancer RCT Pilot Screening Trial: baseline findings from the screening arm provide evidence for the potential implementation of lung cancer screening | Ineligible outcome |
| 5 | Infante 2009 (DANTE) | A randomized study of lung cancer screening with spiral computed tomography: three-year results from the DANTE trial | Ineligible outcome |
| 6 | Kaerlev 2012 (DLCST) | CT-Screening for lung cancer does not increase the use of anxiolytic or antidepressant medication | Ineligible outcome |
| 7 | National Lung Cancer Screening Trial Team (NLST) | Lung Cancer Incidence and Mortality with Extended Follow-up in the National Lung Screening Trial | Ineligible outcome |
| 8 | NCT00006087  (Studts 2006 (JHCS)) | A Randomized Prospective Study Comparing Annual Chest X-Rays to Annual Spiral Chest CT Scanning in Patients at High-Risk for the Development of Lung Cancer | Ineligible outcome |
| 9 | NCT02898441 (Yang 2018 (AME)) | Community-based Early Stage Lung Cancer Screening With Low-dose Computed Tomography in China | Ineligible population |
| 10 | NCT03853967 | Lung Cancer Screening by Low Dose CT Scan in a French Department (DEP KP80) | Ineligible study design |
| 11 | Passiglia, 2021 | Benefits and Harms of Lung Cancer Screening by Chest Computed Tomography: A Systematic Review and Meta-Analysis. | Ineligible study design |
| 12 | Pastorino 2012 (MILD) | Annual or biennial CT screening versus observation in heavy smokers: 5-year results of the MILD trial | Ineligible outcome |
| 13 | Patz 2016 (NLST | Lung cancer incidence and mortality in National Lung Screening Trial participants who underwent low-dose CT prevalence screening: a retrospective cohort analysis of a randomised, multicentre, diagnostic screening trial | Ineligible outcome |
| 14 | Sagawa 2012 (JECS) | A randomized controlled trial on the efficacy of thoracic CT screening for lung cancer in non-smokers and smokers of <30 pack-years aged 50-64 years (JECS study): research design | Ineligible population |
| 15 | Studts 2006 (JHCS) | Validity of self-reported smoking status among participants in a lung cancer screening trial. | Ineligible outcome |
| 16 | Yang 2018 (AME) | Community-based lung cancer screening with low-dose CT in China: Results of the baseline screening. | Ineligible population |
| 17 | Yousaf-Khan 2017 (NELSON) | Final screening round of the NELSON lung cancer screening trial: the effect of a 2.5-year screening interval | Insufficient follow-up |
| **Costs associated with overdiagnosis** | | | |
| ***Identified through Database and registers search*** | | | |
| 1 | Criss 2019 | Cost-effectiveness analysis of lung cancer screening in the United States | Non-European country |
| 2 | Griffin 2020 | Lung cancer screening by low-dose computed tomography: a cost-effectiveness analysis of alternative programmes in the UK using a newly developed natural history-based economic model. | Ineligible population |
| 3 | Haaf 2015 | Performance and Cost-Effectiveness of Computed Tomography Lung Cancer Screening Scenarios in a Population-Based Setting: a Microsimulation Modeling Analysis in Ontario, Canada | Non-European country |
| 4 | Harpaz 2023 | Updated cost-effectiveness analysis of lung cancer screening for Australia, capturing differences in the health economic impact of NELSON and NLST outcomes | Non-European country |
| 5 | Sun 2021 | Determining cost-effectiveness of lung cancer screening in urban Chinese populations using a state-transition Markov model | Non-European country |
| 6 | Wisnivesky 2003 | The cost-effectiveness of low-dose CT screening for lung cancer: preliminary results of baseline screening. | Non-European country |
| ***Identified through citation searching*** | | | |
| 1 | Du 2020 | Cost-effectiveness of lung cancer screening with low-dose computed tomography in heavy smokers: a microsimulation modelling study | Ineligible population |
| 2 | Field 2016B | The UK Lung Cancer Screening Trial: a pilot randomised controlled trial of low-dose computed tomography screening for the early detection of lung cancer | Ineligible population |
| 3 | Jensen 2020 | Direct and indirect healthcare costs of lung cancer CT screening in Denmark: a registry study | Ineligible population |
| 4 | Mahadevia 2023 | Lung cancer screening with helical computed tomography in older adult smokers: a decision and cost-effectiveness analysis. | Non-European country |
| 5 | Nagy 2023 | Shall We Screen Lung Cancer With Low-Dose Computed Tomography? Cost-Effectiveness in Hungary | Ineligible population |
| 6 | Tomonaga 2018 | Cost-effectiveness of low-dose CT screening for lung cancer in a European country with high prevalence of smoking-A modelling study | Ineligible outcome |
