# Supplementary material for: Screening for lung cancer: A systematic review of overdiagnosis and its implications
Source: Mol Oncol. 2025 Nov 11;20(3):611–28. doi: 10.1002/1878-0261.70139 (PMC13042368; doi:10.1002/1878-0261.70139)
Supplement: Supplementary file 6 — Table S3. Overdiagnosis, participation, and contamination in included trials. [file MOL2-20-611-s006.doc]

**Supplementary Table 3: Overdiagnosis, participation, and contamination in included trials**

|  | **Overdiagnosis results** | | **Participation/Contamination** | |
| --- | --- | --- | --- | --- |
| **Study ID** | **Excess incidence** | **Risk of overdiagnosis** | **Participation rates** | **Contamination rates** |
| DANTE^a^ | 36% | 0.26 | NR / Compliance data‐ 1223 (97%) of participants had ≥ 3 CTs, 1184 (94%) had 5 CT scans. | LDCT: 74 extra CT scan, 233 extra CXRs / Control: 68 extra CTs, 209 extra CXRs |
| DLCST | 87% | 0.67 | 95.5% | 20% in the control and 16.5% in the intervention group |
| ITALUNG | -10% | -0.11 | 3y: 80% | NR |
| LUSI | 21% | 0.17 | >90% | 8.7% |
| MILD | 18% | 0.16 | 10y 71% | 1.2% |
| NELSON | 14% | 0.12 | Round 1-3: at least 87.5%; Round 4: 80.7% | NR |
| NLST | 1% | 0.012  NR | LDCT all rounds: 95%; Control: 93% | NR |
| UKLS | 1% | 0.01 | Baseline: 98% | NR |

ID: Identification; LDCT: Low dose computed tomography; CXR: Chest x-ray; DANTE: the Detection and screening of early lung cancer by Novel imaging Technology trial; DLCST: Danish Lung Cancer Screening Trial; ITALUNG: the Italian Lung Cancer Screening trial; LUSI; the Lung Cancer Screening Intervention trial; MILD; the Multicentric Italian Lung Detection trial; NELSON: Nederlands-Leuvens Longkanker Screenings Onderzoek trial; NLST: the National Lung Screening Trial; UKLS: the UK Lung Cancer Screening trial.

a. Recruitment occurred from 2001 to 2006; subjects underwent 5 rounds of screening. Follow up continued until 2012.
